# Neurobiological and behavioural responses of cleaning mutualisms to ocean warming and acidification

**DOI:** 10.1038/s41598-019-49086-0

**Published:** 2019-09-04

**Authors:** José Ricardo Paula, Tiago Repolho, Maria Rita Pegado, Per-Ove Thörnqvist, Regina Bispo, Svante Winberg, Philip L. Munday, Rui Rosa

**Affiliations:** 1 MARE – Marine and Environmental Sciences Centre, Laboratório Marítimo da Guia, Faculdade de Ciências da Universidade de Lisboa, Av. Nossa Senhora do Cabo, 939, 2750-374 Cascais, Portugal; 2 Department of Neuroscience, Physiology Unit, Biomedical Centre (BMC), Uppsala University, Box 593, Uppsala, SE 75124 Sweden; 3 Departamento de Matemática, Centro de Matemática e Aplicações, Faculdade de Ciências e Tecnologia, Universidade Nova de Lisboa, Campus da Caparica, 2829-516 Caparica, Portugal; 4 ARC Centre of Excellence for Coral Reef Studies, James Cook University, Townsville, QLD 4811 Australia

**Keywords:** Behavioural ecology, Social behaviour, Climate-change impacts, Marine biology

## Abstract

Cleaning interactions are textbook examples of mutualisms. On coral reefs, most fishes engage in cooperative interactions with cleaners fishes, where they benefit from ectoparasite reduction and ultimately stress relief. Furthermore, such interactions elicit beneficial effects on clients’ ecophysiology. However, the potential effects of future ocean warming (OW) and acidification (OA) on these charismatic associations are unknown. Here we show that a 45-day acclimation period to OW (+3 °C) and OA (980 μatm pCO_2_) decreased interactions between cleaner wrasses (*Labroides dimidiatus*) and clients (*Naso elegans*). Cleaners also invested more in the interactions by providing tactile stimulation under OA. Although this form of investment is typically used by cleaners to prolong interactions and reconcile after cheating, interaction time and client jolt rate (a correlate of dishonesty) were not affected by any stressor. In both partners, the dopaminergic (in all brain regions) and serotoninergic (forebrain) systems were significantly altered by these stressors. On the other hand, in cleaners, the interaction with warming ameliorated dopaminergic and serotonergic responses to OA. Dopamine and serotonin correlated positively with motivation to interact and cleaners interaction investment (tactile stimulation). We advocate that such neurobiological changes associated with cleaning behaviour may affect the maintenance of community structures on coral reefs.

## Introduction

Mutualisms are ecological interactions that benefit two or more species^1^. Cleaning behaviour is one of the most important mutualistic interactions between fishes in coral reefs^2–4^. Cleanin g directly affects communities structure and health, as the removal of cleaners in small patch reefs decreased fish diversity, recruitment and abundance of both site-attached resident fishes and visitor client fishes^2–4^. While interacting with client fish, some cleaners (e.g. wrasse *Labroides dimidiatus*) can either cooperate (eating parasites, i.e. cleaning) or cheat (eating mucus from clients) which they prefer^5^. Whenever cleaners choose to be dishonest, a conflict arises, typically resulting in an observable “jolt” of the client, in response to the cleaner’s bite^6^. Cleaners can reduce conflict and invest in the quality of the interaction by providing tactile stimulation to their clients (physical contact with client bodies) using their pelvic fins^7^. Additionally, cleaners can increase inspection quality and duration, which enhances the odds of future interactions^8^.

To optimize the output of cooperative interactions, fishes need to adjust their social behaviour according to the available social information (i.e. social competence)^9^. The ability to regulate their social behaviour relies on mechanisms that allow fast and transient behavioural changes, which depend on socially-driven biochemical switching of existing neural networks (e.g. neuromodulators). Monoamines are one major class of neuromodulators and their action in social behaviour as well as their sensitivity to environmental factors, have been extensively documented^10^. They are thought to be involved in control and integration of behaviour and physiological stress response in fish^11^. For instance, cleaner wrasses’ social behaviour is known to be modulated by different monoamines, namely serotonin and dopamine, which affect motivation to interact and interaction quality, respectively^12,13^.

Ocean warming and acidification, caused by the rising concentration of carbon dioxide (CO_2_) in the atmosphere, are predicted to impact fish behaviour and physiology^14^. Sensitivity to ocean acidification acclimation can lead to learning impairments^15^, increased anxiety in rockfish (*Sebastes diploproa*)^16^, disrupted lateralization^17^ and loss of auditory in mulloway larvae (*Argyrosomus japonicus)*^18^ and olfactory responses of orange clown fish (*Amphiprion percula)*^19^. Several of those impairments are assumed to be linked to changes in Cl^−^ ion exchange in γ-aminobutyric acid type A (GABA_A_) receptors, as the administration of gabazine (GABA_A_ antagonist) restored those effects^16,20^. Likewise, ocean warming can impact fish behaviour by increasing activity levels^21^, sensory responsiveness^22^ and prey-predator interactions^23^. In fact, the changes in these climate-change related drivers can unbalance mutualisms towards more exploitative outcomes, where a once-beneficial interaction can become less beneficial or even detrimental^1^.

Although a recent study showed the deleterious impact of extreme environmental perturbations, namely cyclones and bleaching, to cleaner fish abundance and sophistication^24^, there is no knowledge regarding the combined effect of ocean warming and acidification on cleaning mutualisms and how these might affect the respective neuromodulators. Here, we evaluated how simulated end-of-century elevated CO_2_ (~960 µatm, high CO_2_) and warming (+3 °C) scenarios^25^ may affect cooperative cleaning interactions between the cleaner wrasse (*L*. *dimidiatus*) and a client surgeonfish (*Naso elegans*). To analyse the behaviour component of cleaning interactions we measured cleaner fish and client motivation to interact (e.g. number of interactions, the proportion of interactions initiated by cleaners and ratio of client “posing” displays) and interaction quality (e.g. mean interaction duration, number of client jolts and proportion of interactions with tactile stimulation). To study the molecular mechanisms behind such changes in the cleaning interactions, we quantified monoamine levels and metabolites (as a proxy of activity) in three major regions of the fish brain that are normally analysed in social behaviour studies – e.g. forebrain, midbrain and hindbrain^26–28^. Moreover, we investigated how serotoninergic and dopaminergic systems were associated with motivation to engage in cooperation and interaction quality.

## Results

### Behavioural trial

**Figure 1:**
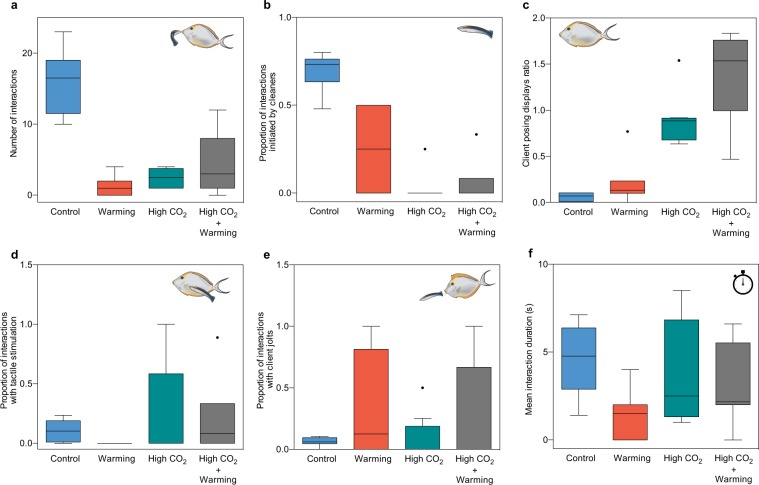
Motivation and quality of cleaning interactions drop under high CO_2_ and warming. Behavioural responses from the interaction trial between the cleaner *Labroides dimidiatus* (n = 31) and the client *Naso elegans* (n = 31). **(a)** Number of interactions, **(b)** proportion of interactions started by cleaners, **(c)** client “posing” displays ratio (number of posings displays divided by time without interaction), (**d)** proportion of interactions with client jolts, **(e)** proportion of interactions with tactile stimulation, **(f)** interaction duration (in seconds). Treatment scenarios are represented by control (present day scenario, temperature = 29 °C, pCO_2_ ~ 400 μatm), warming (temperature = 32 °C, pCO_2_ ~ 400 μatm), high CO_2_ (acidification, temperature = 29 °C, p CO_2_ ~ 960 μatm), high CO_2_ + warming (temperature = 32 °C, p CO_2_ ~ 960 μatm). Box upper and lower edges encloses the interquartile range, the line within each box denotes the median, whiskers extend to the farthest points that are not outliers (i.e. 1.5 × the interquartile range), and the points indicate outliers. Drawings by Catarina Santos.

After 45 days of acclimation, the number of cleaning interactions was significantly decreased under warming (−93%), acidification (−85%) and the interaction of these two stressors (−73%, df = 30, p < 0.001; Fig. 1a, Supplementary Table S2). On average, the proportion of interactions initiated by the cleaners declined under elevated CO_2_ (−66%, df = 26, p < 0.001; Fig. 1b, Supplementary Table S2), but there was no significant effect of warming, or the interaction between stressors. By contrast, the client posing displays ratio significantly increased under high CO_2_ (+84%, df = 30, p < 0.05; Fig. 1c, Supplementary Table S2). While the proportion of cleaning interactions with tactile stimulation significantly increased under high CO_2_ (+15%, df = 26, p < 0.01; Fig. 1d, Supplementary Table S2), cleaners’ dishonesty (proportion of interactions with client jolts) and interaction duration were not affected significantly by either warming or high CO_2_ (df = 26, p > 0.05; Fig. 1e–f, Supplementary Table S2).

### Neurotransmitters concentration

**Figure 2:**
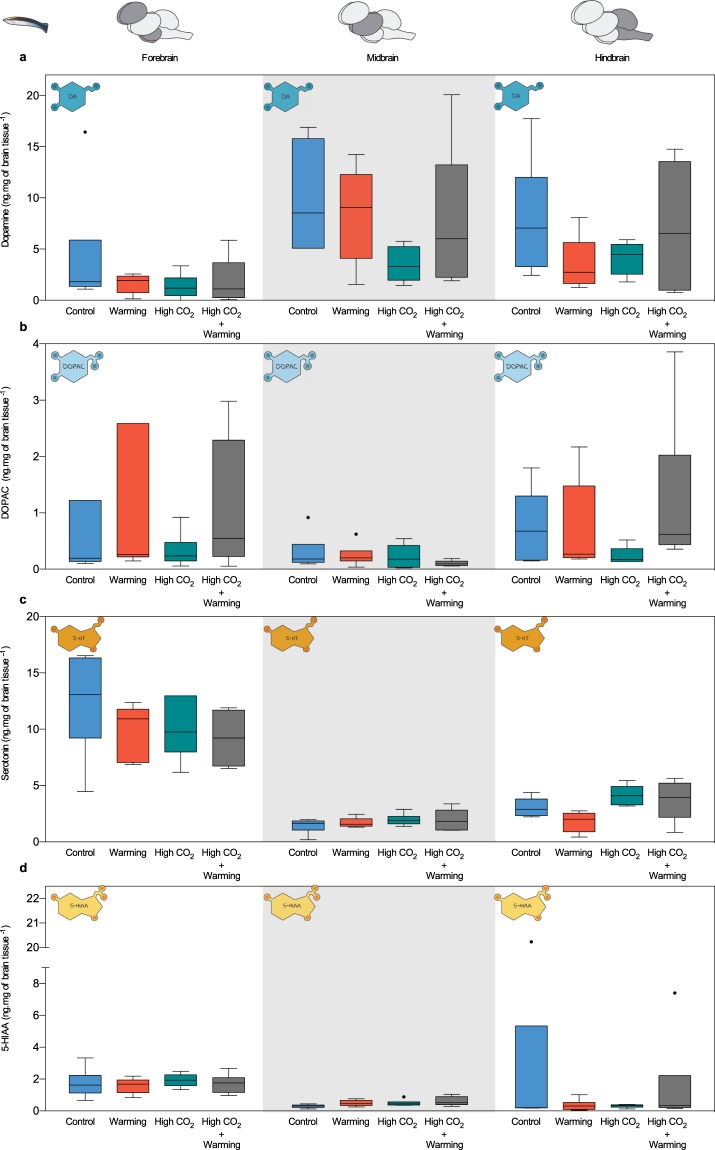
Neurotransmitters and metabolite concentrations in cleaner wrasse (*L*. *dimidiatus*) under high CO_2_ and ocean warming per brain region. (**a**) dopamine concentration (ng.mg of brain tissue^−1^); (**b**) DOPAC concentration (ng.mg of brain tissue^−1^); (**c**) serotonin concentration (ng.mg of brain tissue^−1^); (**d**) 5-HIAA concentration (ng.mg of brain tissue^−1^). Brain regions are divided in forebrain, midbrain and hindbrain. Treatment scenarios are represented by control (present day scenario, temperature = 29 °C, pCO_2_ ~ 400 μatm), warming (temperature = 32 °C, pCO_2_ ~ 400 μatm), high CO_2_ (acidification, temperature = 29 °C, p CO_2_ ~ 960 μatm), high CO_2_ + warming (temperature = 32 °C, p CO_2_ ~ 960 μatm). Y-axis ranges were adjusted allowing better visualisation of concentrations according to different monoamines and fish species. Box upper and lower edges encloses the interquartile range, the line within each box denotes the median, whiskers extend to the farthest points that are not outliers (i.e. 1.5 × the interquartile range), and the points indicate outliers.

In cleaner fish, although dopamine concentration was overall higher in midbrain and hindbrain, a significant interaction of stressors was observed (independently of the brain region), high CO_2_ significantly decreased dopamine only under lower temperatures (−67% in FB, −65% in MB, −48% in HB; df = 23, p < 0.01; Fig. 2a, Supplementary Table S3). On the other hand, DOPAC concentration did not change among treatments (df = 23, p > 0.05, Fig. 2b Supplementary Table S3). Serotonin (5-HT) concentration significantly decreased in the forebrain under high CO_2_ (−19%, df = 23, p < 0.05; Fig. 2c, Supplementary Table S3), while regarding 5-HIAA concentration, there was a significant interaction of stressors in the hindbrain, as high CO_2_ significantly increased 5-HIAA under lower temperature (−91%, df = 23, p < 0.05; Fig. 2d, Supplementary Table S3).

**Figure 3:**
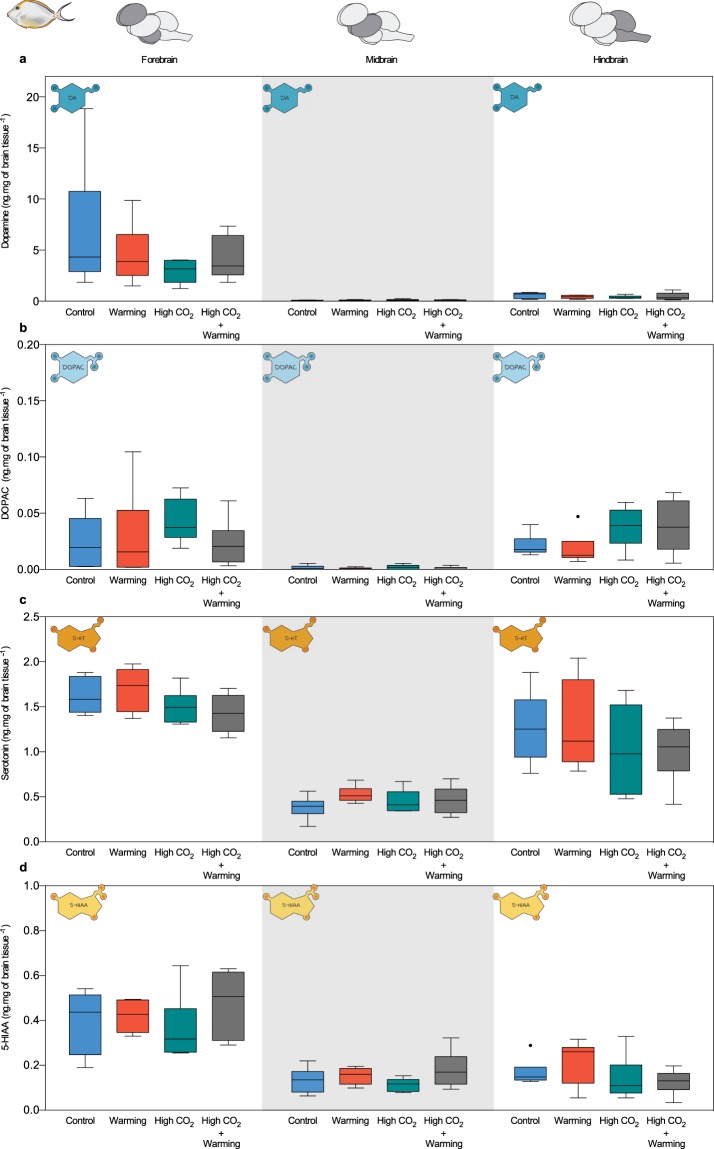
Neurotransmitters and metabolite concentrations in client fish (*N*. *elegans*) under high CO_2_ and ocean warming per brain region. (**a**) dopamine concentration (ng.mg of brain tissue^−1^); (**b**) DOPAC concentration (ng.mg of brain tissue^−1^); (**c**) serotonin concentration (ng.mg of brain tissue^−1^); (**d**) 5-HIAA concentration (ng.mg of brain tissue^−1^). Brain regions are divided in forebrain, midbrain and hindbrain. Treatment scenarios are represented by control (present day scenario, temperature = 29 °C, pCO_2_ ~ 400 μatm), warming (temperature = 32 °C, pCO_2_ ~ 400 μatm), high CO_2_ (acidification, temperature = 29 °C, p CO_2_ ~ 960 μatm), high CO_2_ + warming (temperature = 32 °C, p CO_2_ ~ 960 μatm). Y-axis ranges were adjusted allowing better visualisation of concentrations according to different monoamines and fish species. Box upper and lower edges encloses the interquartile range, the line within each box denotes the median, whiskers extend to the farthest points that are not outliers (i.e. 1.5 × the interquartile range), and the points indicate outliers.

In clients, dopamine concentration (overall higher in the forebrain) was significantly decreased under high CO_2_ in the forebrain (−57%) and hindbrain (−32%), and increased in the midbrain (+77%; df = 23, p < 0.05; Fig. 3a, Supplementary Table S4). Contrarily, DOPAC (df = 23, p > 0.05, Fig. 3a Supplementary Table S4) and serotonin (df = 23, p > 0.05, Fig. 3a Supplementary Table S4) concentrations did not change among treatments. While 5-HIAA concentration significantly decreased in the forebrain under high CO_2_ (−8%, df = 23, p < 0.05; Fig. 2d, Supplementary Table S3).

### Correlation between behaviour and neurotransmitters

The canonical correlation analysis (CCA) between the neurobiological (N) and behaviour (B) sets of variables showed that, in cleaners, 51.9% of the behavioural variability was explained by neurobiological variables. CCA cross-loadings (Supplementary Figs S1-S2) revealed that the first canonical variable (CV1) has a strong negative correlation with proportion of interactions with tactile stimulation (r_NCV1 × Tactile stimulation_ = −0.85) and positive with client jolts (r_NCV1 × client jolts_ = 0.63) and a moderate negative correlation with midbrain 5-HIAA (r_BCV1 × MB-5-HIAA_ = −0.45), and hindbrain serotonin and dopamine concentrations (r_BCV1 × HB-5-HT_ = −0.44, r_BCV1 × HB-DA_ = −0.3). The CCA also showed that CV2 correlates negatively with the proportion of interactions initiated by cleaners (r_NCV2 × interactions initiated by cleaners_ = −0.84) and number of interactions (r_NCV2 × interactions_ = −0.79) and with midbrain dopamine, DOPAC (r_BCV2 × MB-DA_ = −0.6; r_BCV2 × MB-DOPAC_ = −0.36), and hindbrain 5-HIAA concentrations (r_BCV2 × HB-5-HIAA_ = −0.38).

For the client fish, CCA between neurobiological and behavioural variables showed that, on average, 15.7% of the behavioural variability was explained by neurotransmitters concentration. CCA cross-loadings (Supplementary Figs S3-S4) revealed that CV1 has positive correlation with client posing ratio (r_NCV1 × Client posing_ = 0.67) and negative correlation with the midbrain serotonin and 5-HIAA concentrations (r_BCV1 × MB-5-HT_ = −0.29). On the other hand, CV2 correlates negatively with the proportion of interactions with jolts (r_NCV2 × client jolts_ = 0.57), number of interactions (r_NCV2 × interactions_ = −0.51) and with midbrain dopamine (r_BCV2 × MB-DA_ = 0.57).

## Discussion

Fish behaviour and physiology is known to be affected by ocean warming and acidification but their effects on cleaning mutualisms are poorly known^1,14^. Here, we show that both these stressors affect the cooperative cleaning interactions (both at behavioural and neurobiological scales) between the cleaner wrasse *L*. *dimidiatus* and the client fish *N*. *elegans*. The motivation to interact dropped and interaction quality changed under such conditions. Concomitantly, the dopaminergic (in all brain regions) and serotonergic (in the forebrain) systems were significantly altered by these stressors in both partners.

Impaired cleaning interactions can indirectly lead to cascading effects on local fish community structure and abundance. Previous studies suggest that the presence of cleaner wrasses can affect local communities as the experimental removal of cleaners in small patch reefs decreased fish diversity, recruitment and abundance of both site-attached resident fishes and visitor client fishes^2^. Cleaner fish presence is known to affect parasitic gnathiid isopod loads on individuals^3^, and just a single gnathiid ectoparasite can significantly impact fish survival (especially small recruits) as it decreases their swimming performance and metabolic rates and increases mortality rates^29^. Thus, a disruption of cleaning interactions under warming and ocean acidification could indirectly, by a putative increase in clients’ ectoparasite load, affect client fish physiology and, ultimately, survival. The local and individual effects of losing this mutualism could eventually scale up to a community level (i.e. cascading trophic effects^1^).

The motivation to engage in cooperative interactions (number of interactions) was significantly lower under ocean acidification, warming and the combined stressors. Crucially, the cleaners’ motivation to clean declined with acidification, with the proportion of interactions started by cleaners dropping from >75% to <10%. This change in cleaner behaviour was associated with an increase in client posing displays, suggesting that the clients’ motivation to be cleaned had increased.

Clients that experience quality interactions return to the same cleaning station where they were previously inspected, and nearby bystanders choose stations where good quality interactions occur^30^. Thus, investment in the quality of interactions is crucial for cleaners to increase food availability, as they depend solely on cleaning interactions for food^5,30^). Here, cleaner dishonesty (measure as client jolts) and interaction duration were not affected by any stressor. Dishonesty usually increases when cleaners are interacting with resident clients that cannot choose among different cleaning stations^31^. As cleaners were deprived of clients during the whole exposure period, the clients could have been perceived as visitors, leading to the low number of interactions with jolts. Another possible explanation is related with the low number of interactions observed under the different stressors, as cleaners might have lacked the opportunity or motivation to cheat during the behavioural trial. The opposite occurred under acidification, as tactile stimulation events increased under high CO_2._ Cleaners use tactile stimulation to negotiate when the outcome of an interaction is not certain or to prolong interactions after a cheating event. Contrary to predictions, the cleaners’ dishonesty levels remained unaffected by ocean acidification conditions. As tactile stimulation is incompatible with foraging, and it is a costly form of negotiation^32^, ocean acidification might be modulating cleaner wrasse anticipation/perception regarding predicted rewards and cost in an interaction, as if the client was more likely to terminate the interaction. The observed effects of ocean warming and acidification on motivation to engage in cleaning interactions and interaction quality could lead to mutualism loss by: (i) reduction of interactions, ceasing the exchange of rewards between partners; and (ii) loss of negotiation, leading to unfruitful interactions for at least one partner (cleaner)^1,33^.

Here we also showed that ocean warming and acidification altered monoamine concentrations in the different brain regions. So far social behaviour in fishes, including the present species pair, was described to be modulated by different monoaminergic concentrations in different brain regions^26–28^. As expected, these stressors had distinct interspecific effects and differences among the studied brain region. Under ocean acidification, cleaners’ dopamine concentration was overall lower, and no changes were observed in its metabolite DOPAC concentration. Interestingly, hindbrain dopamine concentration was correlated with tactile stimulation. These findings are aligned with a previous study where dopamine disruption was shown to affect directly tactile stimulation without affecting cleaner honesty^13^. In such study, the authors described that ﻿blocking dopamine receptors induces cleaners to initiate more interactions with clients, contrarily, we observed less interactions and less motivation to interact together with a decrease in dopamine concentration under high CO_2_. Moreover midbrain dopamine concentration was correlated number of interactions and cleaners’ motivation to interact. ﻿Here, since we observed the effect after the lack of interactions, it was logical to expect low levels of dopamine related with lower rewards or decreased likelihood to obtain food^13,34^. Since dopamine is responsible for modulating cleaners’ perception of rewards and risks, influencing their capacity to manipulate clients within an interaction, our findings suggest a potential mechanistic effect of high CO_2_ on the dopaminergic system with consequences on reward and risk perception.

In clients, dopamine concentration also decreased overall under high CO_2_ and, in midbrain, was correlated with the number of interactions which, as with cleaners, could be related with lower rewards due to the lack of interactions^26^. Since dopaminergic activation can be: (i) driven by the perception of the interactions, and (ii) related to species-specific social behaviours across vertebrates (as aggression and courtship), we argue that ocean acidification may lead to dopamine-induced impairment of clients’ perception of rewards, as a visual stimulus (cleaner’s presence) is perceived without achieving an interaction (reward). Together, the across species effect on the dopaminergic system suggests dopamine as a potential neurobiological system by which high CO_2_ impairs behaviour although further pharmacological studies are needed to confirm this. Dopamine was not affected by CO_2_ when cleaners were exposed to higher temperatures this could suggest an antagonistic interaction of both stressors and the presence of a cross-tolerance mechanism^35^.

In the serotonergic system, we observed decrease in cleaners’ forebrain serotonergic concentration and overall 5-HIAA increase along with a lower motivation to interact under ocean acidification. The serotonin blockade is known to disrupt motivation for cleaning interactions^12^ and forebrain serotonergic activity can regulate and be regulated by social interactions^26^. Therefore, we suggest that CO_2_ could affect serotonin function disrupting the motivation to interact. In clients serotonin concentration was not affected by any stressor, and 5-HIAA decreased in the forebrain under high CO_2_. A previous study^12^ had already shown that serotonin may not affect clients’ direct interactions with cleaners, and thus motivation to interact is not equally modulated in cleaners and clients. It is worth noting that contrary to a recent study in cod, which found a higher serotonergic activity under ocean warming^36^, the serotoninergic system was not affected by warming alone in both fishes. We argue that both mutualistic partners live in a relatively more stable temperature environment than temperate and sub-arctic cod species, and therefore they may lack the ability of serotonergic modulation of respiration observed in cod, but further investigation is needed to test this hypothesis. The metabolite 5-HIAA was also not affect by CO_2_ when cleaners were exposed to higher temperatures again suggesting an antagonistic interactions of stressors an cross-tolerance mechanisms^35^.

It is worth noting that GABAergic neurotransmission is the major system known to be affected by ocean acidification and one cannot discard its effects and possible GABAergic interactions with dopamine and serotonin^16,20,37^. Under elevated CO_2_, the equilibrium potential for Cl^−^ is disrupted due to a decrease in plasma Cl^−^ concentration to maintain charge balance (due to H^+^ excretion to counteract acidosis). When GABA binds to the normally inhibitory GABA_A_ receptor channel opening leads to net Cl^−^ movement out of the neuron, causing membrane depolarization and increasing excitation of neural pathways^38^. This process was described in both fish and invertebrates – e.g. mollusks^39,40^. The GABAergic system is known to be linked with both dopaminergic and serotonergic systems, as GABA_A_ receptor activation in GABAergic interneurons can reduce serotonergic response^41^ and dopamine neurons can exert a strong inhibitory influence through activation of GABA_A_ receptors^42^. Therefore, the described effect of CO_2_ in GABAergic receptors, and their relation with serotonin and dopamine can play a role in the potential mechanism of high CO_2_ disruption of cooperative cleaning behaviour.

In conclusion, we show that cooperative cleaning interactions, a key mutualism in coral reefs, are disrupted by ocean warming and acidification conditions. This disruption could potential lead to mutualism breakdown if: (1) this mutualism shift to antagonism, (2) one of the partner switches to novel partners or (3) both partners abandon the mutualistic interaction^1,33^. Mutualism integrity is affected mainly by a lower motivation to engage in interactions and lack of proper perception of interaction quality by cleaners, by disrupting their ability to negotiate (under elevated CO_2_). We suggest that CO_2_ could interact (directly or indirectly) with: (i) dopamine, a new potential neurobiological system of CO_2_ behaviour impairment, damaging cleaners’ and clients’ perception and ability to negotiate and (ii) serotonin modulating motivation for interaction, although further pharmacological studies are required to support this hypothesis. Unravelling the interspecific behavioural effect of ocean warming and acidification with neurobiological links is a priority for future research, as it would provide the opportunity to better understand possible behavioural impairments at the community level. Thus, we propose that a climate change induced neurobiological and behavioural disruption of cleaning mutualisms will significantly affect the structure of local communities of coral reefs.

## Methods

### Acclimation conditions

We used cleaner wrasses *Labroides dimidiatus* (n = 32; size 5.4 ± 0.6 cm) and surgeon fish *Naso elegans* (n = 32, size 6.9 ± 1.1 cm), a frequent client of cleaner wrasses known to be easily adapted to laboratory conditions^43^. These species have been used in behavioural and neurobiological studies and exhibit fully their cooperative cleaning interactions in aquaria even without the presence of parasites. Both were collected by local fishermen, using hand nets and barrier nets, between November and December 2014 in the Maldives and transported by TMC Iberia to the aquatic facilities of Laboratório Marítimo da Guia (Cascais, Portugal). To avoid possible interactions between parasitation levels, parasite-fish interaction and parasite responses to ocean warming and acidification treatments fish were deparasitized with a five-minute freshwater bath on arrival^44^. Fish were also laboratory acclimated for 5 days at seawater conditions similar to the collection site: salinity = 35 ± 0.5, temperature 29 °C (Maldives 2013–2014 average SST, NOAA^45^), pH 8.1 and pCO_2_ ~400 ppm (2014 BOBOA Ocean Acidification mooring, NOAA^46^). Each fish was exposed for 45 days (to one of the four following experimental treatments) in separated individual tanks (i.e. 8 *L*. *dimidiatus* and 8 *N*. *elegans* per treatment, 32 *L*. *dimidiatus* and 32* N*. *elegans* in total; a total of 64 tanks): (1) present day scenario (control) (29 °C, pH 8.1, pCO2 ~ 400 ppm), (2) warming scenario (32 °C, pH 8.1, pCO2 ~400 ppm), (3) high CO_2_ – acidification (29 °C, pH 7.7, pCO2 ~960 ppm) and (4) high CO_2_ + warming (32 °C, pH 7.7, pCO2 ~960 ppm), following IPCC’s RCP scenario 8.5^25^ (see Table S1 for water chemistry summary). During the exposure period one fish from each species died in the warming scenario.

In each treatment, we used flow-through aquatic systems to maintain correct levels of total alkalinity, dissolved inorganic carbon and pH. Natural seawater (NSW) was pumped from the sea into a 5 m^3^ seawater storage tank. From the storage tank, NSW was filtered (0.35 μm) and UV-irradiated (Vecton 300, TMC Iberia, Portugal) before being supplied to mixing and experimental tanks. Experimental tanks were kept under a photoperiod of 12 h/12 h (light/dark cycle). Ammonia and nitrate levels were daily checked using colorimetric tests (Salifert Profi Test, Holland). Levels of pH were monitored and automatically adjusted every 2 seconds (Profilux 3.1 N, GLH, Germany), downregulated by direct injection of certified CO_2_ gas (Air Liquide, Portugal) and upregulated through aeration with CO_2_ filtered atmospheric air (soda lime, Sigma-Aldrich) in mixing tanks. Seawater temperature was regulated using chillers (Frimar, Fernando Ribeiro Lda, Portugal) and submerged heaters (300 W, TMC-Iberia, Portugal). We used additional handheld equipment to complement the automatic systems with a manual daily monitoring of seawater temperature (TFX 430 thermometer, WTW GmbH, Germany), salinity (V2 refractometer, TMC Iberia, Portugal) and pH (826 pH mobile, Metrohm, Germany). We quantified pH using a pH meter connected to a glass electrode (Schott IoLine, Si analytics, ±0.001), calibrated with TRIS-HCl (TRIS) and 2-aminopyridine-HCl (AMP) seawater buffers. Seawater carbonate system speciation was calculated twice a week from total alkalinity (spectrophotometrically at 595 nm) and pH measurements^40^. Bicarbonate and pCO_2_ values were calculated using CO2SYS software. Seawater parameters of different experimental setups are summarized in Supplementary Table S5.

### Behavioural observations

Following 45 days of acclimation, both cleaners and clients were fasted for 24 hours. After fasting, pairs composed of one cleaner *L*. *dimidiatus* and one client *N*. *elegans* interacted for 30 minutes (between 08:00–12:00) in observation tanks (3 isolated aquaria: 30 cm × 40 cm × 40 cm) with the same water parameters as the experimental treatments. Fish were acclimatized to the observation tank for 15 minutes prior to the experiment separated by an opaque acrylic partition. During the observation period, the experimenter raised the partition, left the room, and the behavioural trial was recorded for 30 minutes by a video camera (Canon Legria HFR56) positioned frontally to the observation tank wall. Before each trial water was renovated and the tank was cleaned to avoid possible chemical cues. To characterize cleaner and client motivation to interact, we measured the number of interactions, the proportion of interactions initiated by cleaners and ratio of client “posing” displays (i.e. client “posing displays/time of no interaction; “posing” displays are conspicuous signals used by clients seeking cleaning interactions from cleaners^47^). Cleaning interaction quality was determined using mean interaction duration, number of client jolts (jolt is a conspicuous signal that indicates cheating^48^) and proportion of interactions with tactile stimulation (cleaners use tactile stimulation of clients with their pectoral fins to manipulate them as this behaviour has been shown to reduce stress levels and can prolong interaction duration^4,7^). All data were analysed per trial (30 minute observation).

### Brain sampling and quantification of brain monoamines and metabolites

To avoid monoamine degradation during the brain macro-dissection and to keep the time of sampling after the interactions as homogeneous, cleaner and client fishes were sacrificed immediately after an overdose of tricaine solution (MS222, Pharmaq; 250 mg/L) and the spinal cord sectioned. The brain was macrodissected under a stereoscope (Leica S6D) into three regions: forebrain (olfactory bulbs + telencephalon + diencephalon, FB), midbrain (optic tectum, MB), and hindbrain (cerebellum + brainstem, HB). Immediately after collection brain tissue was placed on dry ice and stored at −80 °C until analysis.

Frozen brain regions from 24 interacting pairs (48 fish, 12 per treatment) were homogenized in 4% (w/v) ice-cold perchloric acid containing 100 ng/ml 3,4-dihydroxybenzylamine (DHBA, the internal standard) using a Sonifier cell disruptor B-30 (Branson Ultrasonics, Danbury, CT, USA) and were immediately placed on dry ice. Subsequently, the homogenized samples were thawed and centrifuged at 21,000 × g for 10 min at 4 °C. The supernatant was used for high-performance liquid chromatography with electrochemical detection (HPLC-EC), analyzing the monoamines dopamine (DA) and serotonin (5-HT, 5-hydroxytryptamine), the DA metabolite DOPAC (3,4-dihydroxyphenylacetic acid), and the 5-HT metabolite 5-HIAA (5-hydroxy indole acetic acid), as described by Teles *et al*.^26^. In brief, the HPLC–EC system consisted of a solvent delivery as system model 582 (ESA, Bedford, MA, USA), an auto injector Midas type 830 (Spark Holland, Emmen, the Netherlands), a reverse phase column (Reprosil-Pur C18-AQ 3 μm, 100 mm × 4 mm column, Dr. Maisch HPLC GmbH, Ammerbuch-Entringen, Germany) kept at 40 °C and an ESA 5200 Coulochem II EC detector (ESA, Bedford, MA, USA) with two electrodes at reducing and oxidizing potentials of −40 mV and +320 mV. A guarding electrode with a potential of +450 mV was employed before the analytical electrodes to oxidize any contaminants. The mobile phase consisted of 75 mM sodium phosphate, 1.4 mM sodium octyl sulphate and 10 μM EDTA in deionized water containing 7% acetonitrile brought to pH 3.1 with phosphoric acid. Samples were quantified by comparison with standard solutions of known concentrations. To correct for recovery DHBA was used as an internal standard using HPLC software ClarityTM (DataApex Ltd., Prague, Czech Republic).

### Statistical analysis

Data exploration was performed according to Zuur *et al*.^49^, which promotes a protocol for data exploration. Behavioural data was analysed using Generalized Linear Models (GLM) using pCO_2_ and temperature as covariates, according to Zuur and Ieno^50^. In these models, Gaussian distribution was used for continuous data (interaction duration and client posing ratio); negative binomial distribution for count data (number of interactions, since we observed overdispersion of Pearson residuals using Poisson distribution) and binomial distribution for proportions (proportion of interactions started by cleaners, proportion of interactions with tactile stimulation and proportion of interactions with client jolts). For neurobiological data analysis, we used a Generalized Linear Mixed Model framework with individual identity as a random effect, pCO_2_, temperature and brain region as covariates and Gamma distribution. Selection for best model was made using Akaike Information Criterion (AIC). Model assumptions, namely independence and absence of residual patterns, were verified by plotting residuals against fitted values and each covariate in the model. A canonical correlation analysis was performed to explore the correlation between two paired variable sets, behaviour variables and neurobiological variables for each species. Statistical analysis was performed in R^51^ and data exploration and model validation used the HighstatLibV10 R library from Highland Statistics^52^.

### Ethical note

Research was conducted under approval of Faculdade de Ciências da Universidade de Lisboa animal welfare body (ORBEA – Statement 01/2017) and Direção-Geral de Alimentação e Veterinária (DGAV – Permit 2018-05-23-010275) in accordance with the requirements imposed by the Directive 2010/63/EU of the European Parliament and of the Council of 22 September 2010 on the protection of animals used for scientific purposes.

## Supplementary information

Supplementary Information

## Data Availability

Data supporting this article is available in the repository figshare 10.6084/m9.figshare.7235192.
