# Supplementary material for: Neurobiological and behavioural responses of cleaning mutualisms to ocean warming and acidification
Source: Sci Rep. 2019 Sep 4;9:12728. doi: 10.1038/s41598-019-49086-0 (PMC6726634; doi:10.1038/s41598-019-49086-0)
Supplement: Supplementary file 1 — Supplementary Information [file 41598_2019_49086_MOESM1_ESM.pdf]

Supplementary information

Neurobiological and behavioural responses of cleaning mutualisms to ocean warming and acidification

José Ricardo Paula[1*], Tiago Repolho[1], Maria Rita Pegado[1], Per-Ove Thörnqvist[2], Regina Bispo[3], Svante Winberg[2], Philip L. Munday[4], Rui Rosa[1]

[1] MARE – Marine and Environmental Sciences Centre, Laboratório Marítimo da Guia, Faculdade de Ciências da Universidade de Lisboa, Av. Nossa Senhora do Cabo, 939, 2750-374 Cascais, Portugal

[2] Department of Neuroscience, Physiology Unit, Biomedical Centre (BMC), Uppsala University, Box 593, Uppsala SE-75124, Sweden

[3] Departamento de Matemática, Centro de Matemática e Aplicações, Faculdade de Ciências e Tecnologia, Universidade Nova de Lisboa, Campus da Caparica, 2829-516 Caparica, Portugal

[4] ARC Centre of Excellence for Coral Reef Studies, James Cook University, Townsville, QLD 4811, Australia

* Corresponding author: José Ricardo Paula; Telephone: +351 214869211; E-mail: jrpaula@fc.ul.pt;

Table of Contents

Table S1. Seawater physicochemical parameters in all experimental setups. 2

Table S2. GLM analysis of the behavioural variables 3

Table S3. GLMM analysis of the neurobiological variables measured in cleaner fish. 4

Table S4. GLMM analysis of the neurobiological variables measured in client fish. 6

Figure S1-S2. Canonical correlation analysis between in cleaner fish. 8

Figure S3-S4. Canonical correlation analysis in client fish. 9

TABLE S1. Seawater physicochemical parameters in all experimental setups.

	Control	High CO2	Warming	Warming + High CO2
Measured				
**Temperature(ºC)**	29.0 ± 0.3	29.0 ± 0.3	32.0 ± 0.6	32.0 ± 0.6
**pH**	8.04 ± 0.01	7.73 ± 0.01	8.04 ± 0.01	7.73 ± 0.02
**AT (µmol/kgSW)**	2388.4 ± 32.9	2398.9 ± 44.3	2392.0 ± 44.8	2392.3 ± 48.1
**Salinity (ppt)**	35	35	35	3.50E+01
Calculated				
**CT (µmol/kgSW)**	2043.0 ± 32.9	2224.1 ± 42.8	2017.2 ± 40.2	2197.6 ± 46.6
**pCO2 (µatm)**	409.0 ± 12.3	963.6 ± 37.0	404.8 ± 13.8	961.6 ± 45.7
**Ω Arg**	4.0 ± 0.1	2.3 ± 0.1	4.4 ± 0.1	2.5 ± 0.1

Salinity, pH and temperature were measured daily and averaged over the whole experimental period. The combination of total alkalinity (AT) and pHT (pH total scale) was used to calculate carbonate system parameters [pCO2 (carbon dioxide partial pressure), CT (total inorganic carbon) and Ω Arg (aragonite saturation state)]. Values are represented as mean ± standard deviation

TABLE S2. Generalized Linear Models (GLM) analysis of the behavioural variables (number of interactions; proportion of interactions started by cleaners; client posing displays ratio; proportion of interactions with tactile stimulation; proportion of interactions with client jolts; interaction duration) measured during the behavioural trial between the cleaner fish *Labroides dimidiatus* and the client *Naso elegans*.

Model: GLM (Negative Binomial)	*Response variable: Number of interactions*			
Final model term(s): Principal effects of CO_2, temperature and interaction between stressors				
	Estimate	Std. Error	Estimate/ Std. Error	p
(Intercept)	2.77	0.15	18.72	**< 2e-16**
Warming	-2.64	0.4	-6.54	**6.40E-11**
High CO2	-1.91	0.3	-6.41	**1.50E-10**
Warming × High CO2	3.22	0.52	6.16	**7.20E-10**
Model: GLM (Binomial)	*Response variable: Proportion of interactions started by cleaners*			
Final model term(s): Principal effects of CO_2				
	Estimate	Std. Error	Estimate/ Std. Error	p
(Intercept)	0.67	0.18	3.7	**2.10E-04**
High CO2	-3.48	0.62	-5.61	**2.10E-08**
Model: GLM (Gaussian)	*Response variable: Client posing displays ratio*			
Final model term(s): Principal effects of CO2, temperature and interaction between stressors				
	Estimate	Std. Error	Estimate/ Std. Error	p
(Intercept)	0.06	0.11	0.56	0.58
Warming	0.16	0.16	0.99	0.33
High CO2	0.84	0.15	5.42	**9.86E-06**
Warming × High CO2	0.31	0.22	1.37	0.18
Model: GLM (Binomial)		*Response variable: Proportion of interactions with tactile stimulation*		
Final model term(s): Principal effects of CO2				
	Estimate	Std. Error	Estimate/ Std. Error	p
(Intercept)	-2.17	0.28	-7.67	**1.70E-14**
High CO2	1.24	0.42	2.97	**2.90E-03**
Model: GLM (Binomial)	*Response variable: Proportion of interactions with client jolts*			
Final model term(s): Principal effects of temperature				
	Estimate	Std. Error	Estimate/ Std. Error	p
(Intercept)	-2.71	0.37	-7.42	**1.20E-13**
Warming	1.03	0.73	1.42	0.16
High CO2	1.61	0.89	1.80	0.07
Warming × High CO2	-1.95	1.22	-1.60	0.11
Model: GLM (Gaussian)	*Response variable: Interaction duration*			
Final model term(s): Principal effects of CO2, temperature and interaction between stressors				
	Estimate	Std. Error	Estimate/ Std. Error	p
(Intercept)	4.63	0.81	5.72	**7.90E-06**
Warming	-2.31	1.40	-1.65	0.11
High CO2	-1.05	1.14	-0.92	0.37
Warming × High CO2	2.34	1.83	1.28	0.22

Numbers in bold p<0.05

TABLE S3. **Generalized Linear Mixed Models (GLMM) analysis of the neurobiological variables (dopamine, serotonin, DOPAC and 5-HIAA concentration) measured in the cleaner fish *L. dimidiatus*.**

Model: GLMM (Gamma)	*Response variable: Dopamine concentration*			
Final model term(s): Principal effects of CO_2, temperature, brain region and interaction between CO2 and temperature				
Reference level (Brain region): FB	Estimate	Std. Error	Estimate/ Std. Error	p
(Intercept)	1.14	0.27	4.22	**2.50E-05**
High CO2	-0.89	0.30	-2.99	**2.80E-03**
Warming	-0.56	0.30	-1.85	0.06
Brain region (FBvsHB)	1.00	0.24	4.16	**3.20E-05**
Brain region (FBvsMB)	1.26	0.24	5.17	**2.30E-07**
High CO2 x Warming	1.13	0.42	2.71	**0.01**
Reference level (Brain region): MB	Estimate	Std. Error	Estimate/ Std. Error	p
(Intercept)	2.40	0.26	9.31	**2.00E-16**
High CO2	-0.89	0.30	-2.99	**2.80E-03**
Warming	-0.56	0.30	-1.85	0.06
Brain region (MBvsFB)	-1.26	0.24	-5.17	**2.30E-07**
Brain region (MBvsHB)	-0.27	0.24	-1.13	**0.26**
High CO2 x Warming	1.13	0.42	2.71	**0.01**
Model: GLMM (Gamma)	*Response variable: DOPAC concentration*			
Final model term(s): Principal effects of CO_2, temperature, brain region and interaction between them.				
Reference level (Brain region): FB	Estimate	Std. Error	Estimate/ Std. Error	p
(Intercept)	1.92	0.58	3.30	**9.60E-04**
High CO2	1.40	1.14	1.24	0.22
Warming	-0.32	0.73	-0.44	0.66
Brain region (FBvsHB)	0.06	0.38	0.15	0.88
Brain region (FBvsMB)	1.69	1.06	1.60	0.11
High CO2 x Warming	-1.59	1.32	-1.20	0.23
High CO2 x Brain region (FBvsHB)	0.97	1.61	0.60	0.55
High CO2 x Brain region (FBvsMB)	-0.36	1.96	-0.18	0.85
Warming x Brain region (FBvsHB)	0.30	0.54	0.55	0.58
Warming x Brain region (FBvsMB)	1.01	1.65	0.61	0.54
High CO2 x Warming x Brain region (FBvsHB)	-1.39	1.68	-0.82	0.41
High CO2 x Warming x Brain region (FBvsMB)	5.88	3.78	1.56	0.12
Reference level (Brain region): MB	Estimate	Std. Error	Estimate/ Std. Error	p
(Intercept)	3.61	1.07	3.36	**7.70E-04**
High CO2	1.04	1.77	0.59	0.56
Warming	0.69	1.68	0.41	0.68
Brain region (MBvsFB)	-1.69	1.06	-1.60	0.11
Brain region (MBvsHB)	-1.63	1.07	-1.53	0.13
High CO2 x Warming	4.29	3.70	1.16	0.25
High CO2 x Brain region (MBvsFB)	0.36	1.96	0.18	0.85
High CO2 x Brain region (MBvsHB)	1.33	2.14	0.62	0.53
Warming x Brain region (MBvsFB)	-1.01	1.65	-0.61	0.54
Warming x Brain region (MBvsHB)	-0.72	1.67	-0.43	0.67
High CO2 x Warming x Brain region (MBvsFB)	-5.88	3.78	-1.56	0.12
High CO2 x Warming x Brain region (MBvsHB)	-7.26	3.89	-1.87	0.06
Model: GLMM (Gamma)	*Response variable: Serotonin concentration*			
Final model term(s): Principal effects of CO2, brain region and interaction between them.				
Reference level (Brain region): FB	Estimate	Std. Error	Estimate/ Std. Error	p
(Intercept)	0.0896	0.0103	8.69	**2.00E-16**
High CO2	0.01	0.02	0.86	0.39
Brain region (FBvsHB)	0.31	0.05	6.32	**2.50E-10**
Brain region (FBvsMB)	0.54	0.07	7.38	**1.50E-13**
High CO2 x Brain region (FBvsHB)	-0.16	0.06	-2.75	**0.01**
High CO2 x Brain region (FBvsMB)	-0.14	0.09	-1.46	0.14
Reference level (Brain region): MB	Estimate	Std. Error	Estimate/ Std. Error	p
(Intercept)	0.63	0.07	8.69	**2.00E-16**
High CO2	-0.12	0.09	-1.33	0.18
Brain region (MBvsFB)	-0.54	0.07	-7.38	**1.50E-13**
Brain region (MBvsHB)	-0.23	0.09	-2.63	**0.01**
High CO2 x Brain region (MBvsFB)	0.14	0.09	1.46	0.14
High CO2 x Brain region (MBvsHB)	-0.02	0.11	-0.21	0.83
Model: GLMM (Gamma)	*Response variable: 5-HIAA concentration*			
Final model term(s): Principal effects of CO2, temperature, brain region and interaction between them.				
Reference level (Brain region): FB	Estimate	Std. Error	Estimate/ Std. Error	p
(Intercept)	0.81	0.20	3.95	**7.70E-05**
High CO2	-0.20	0.26	-0.77	0.44
Warming	-0.09	0.28	-0.33	0.74
Brain region (FBvsHB)	-0.13	0.13	-0.98	0.33

Brain region (FBvsMB)	2.55	0.91	2.81	**4.90E-03**
High CO2 x Warming	0.26	0.39	0.65	0.51
High CO2 x Brain region (FBvsHB)	2.74	0.88	3.10	**1.90E-03**
High CO2 x Brain region (FBvsMB)	-1.10	1.07	-1.03	0.30
Warming x Brain region (FBvsHB)	2.21	0.78	2.85	**4.40E-03**
Warming x Brain region (FBvsMB)	-1.19	1.07	-1.11	0.27
High CO2 x Warming x Brain region (FBvsHB)	-4.76	1.19	-4.01	**6.00E-05**
High CO2 x Warming x Brain region (FBvsMB)	0.68	1.29	0.53	0.60
Reference level (Brain region): MB	Estimate	Std. Error	Estimate/ Std. Error	p
(Intercept)	3.36	0.90	3.73	**1.90E-04**
High CO2	-1.31	1.06	-1.23	0.22
Warming	-1.29	1.06	-1.21	0.23
Brain region (MBvsFB)	-2.55	0.91	-2.81	**4.89E-03**
Brain region (MBvsHB)	-2.68	0.90	-2.97	**2.99E-03**
High CO2 x Warming	0.94	1.28	0.73	0.46
High CO2 x Brain region (MBvsFB)	1.10	1.07	1.03	0.30
High CO2 x Brain region (MBvsHB)	3.84	1.36	2.82	**4.84E-03**
Warming x Brain region (MBvsFB)	1.19	1.07	1.11	0.27
Warming x Brain region (MBvsHB)	3.41	1.30	2.63	**0.01**
High CO2 x Warming x Brain region (MBvsFB)	-0.68	1.30	-0.53	0.60
High CO2 x Warming x Brain region (MBvsHB)	-5.44	1.71	-3.17	**1.51E-03**

Numbers in bold p<0.05

TABLE S4. Generalized Linear Mixed Models (GLMM) analysis of the neurobiological variables (dopamine, serotonin, DOPAC and 5-HIAA concentration) measured in the client fish *Naso elegans*.

Model: GLMM (Gamma)	*Response variable: Dopamine concentration*			
Final model term(s): Principal effects of CO_2, temperature, brain region and interaction between CO2 and temperature				
Reference level (Brain region): FB	Estimate	Std. Error	Estimate/ Std. Error	p
(Intercept)	0.18	0.05	3.73	**1.90E-04**
High CO2	0.17	0.09	2.01	**0.04**
Warming	0.06	0.07	0.89	0.37
Brain region (FBvsHB)	1.87	0.22	8.43	**2.00E-16**
Brain region (FBvsMB)	10.50	1.11	9.42	**2.00E-16**
High CO2 x Warming	-0.16	0.11	-1.38	0.17
Reference level (Brain region): MB	Estimate	Std. Error	Estimate/ Std. Error	p
(Intercept)	10.68	1.12	9.58	**<2E-16**
High CO2	0.17	0.09	2.01	**0.04**
Warming	0.06	0.07	0.89	0.37
Brain region (MBvsFB)	-10.50	1.11	-9.42	**<2E-16**
Brain region (MBvsHB)	-8.63	1.14	-7.60	**3.00E-14**
High CO2 x Warming	-0.16	0.11	-1.38	0.17
Model: GLMM (Gamma)	*Response variable: DOPAC concentration*			
Final model term(s): Principal effects of CO_2, temperature, brain region and interaction between them.				
Reference level (Brain region): FB				
(Intercept)	-3.77	0.49	-7.74	**9.90E-15**
High CO2	0.62	0.66	0.94	0.35
Warming	0.24	0.66	0.36	0.72
Brain region (FBvsHB)	-0.08	0.66	-0.12	0.91
Brain region (FBvsMB)	-2.73	0.66	-4.14	**3.50E-05**
High CO2 x Warming	-0.85	0.91	-0.93	0.35
High CO2 x Brain region (FBvsHB)	-0.06	0.91	-0.06	0.95
High CO2 x Brain region (FBvsMB)	-0.36	0.91	-0.39	0.70
Warming x Brain region (FBvsHB)	-0.40	0.91	-0.44	0.66
Warming x Brain region (FBvsMB)	-0.79	0.91	-0.86	0.39
High CO2 x Warming x Brain region (FBvsHB)	1.04	1.27	0.82	0.41
High CO2 x Warming x Brain region (FBvsMB)	0.52	1.27	0.41	0.68
Reference level (Brain region): MB	Estimate	Std. Error	Estimate/ Std. Error	p
(Intercept)	-6.50	0.45	-14.61	**2.00E-16**
High CO2	0.26	0.63	0.42	0.67
Warming	-0.55	0.63	-0.88	0.38
Brain region (MBvsFB)	2.73	0.66	4.14	**3.50E-05**
Brain region (MBvsHB)	2.65	0.63	4.22	**2.50E-05**
High CO2 x Warming	-0.33	0.89	-0.37	0.71
High CO2 x Brain region (MBvsFB)	0.36	0.91	0.39	0.70
High CO2 x Brain region (MBvsHB)	0.30	0.89	0.34	0.74
Warming x Brain region (MBvsFB)	0.79	0.91	0.86	0.39
Warming x Brain region (MBvsHB)	0.39	0.89	0.43	0.66
High CO2 x Warming x Brain region (MBvsFB)	-0.52	1.27	-0.41	0.68
High CO2 x Warming x Brain region (MBvsHB)	0.52	1.26	0.41	0.68
Model: GLMM (Gamma)	*Response variable: Serotonin concentration*			
Final model term(s): Principal effects of CO_2, and brain region				
Reference level (Brain region): FB	Estimate	Std. Error	Estimate/ Std. Error	p
(Intercept)	0.51	0.07	7.33	**2.30E-13**
High CO2	-0.12	0.08	-1.60	0.11
Brain region (FBvsHB)	-0.33	0.08	-4.27	**2.00E-05**
Brain region (FBvsMB)	-1.24	0.08	-16.25	**2.00E-16**
Reference level (Brain region): MB	Estimate	Std. Error	Estimate/ Std. Error	p
(Intercept)	-0.73	0.07	-10.40	**<2E-16**
High CO2	-0.12	0.08	-1.60	0.11
Brain region (MBvsFB)	1.24	0.08	16.20	**<2E-16**
Brain region (MBvsHB)	0.91	0.08	12.00	**<2E-16**
Model: GLMM (Gamma)	*Response variable: 5-HIAA concentration*			
Final model term(s): Principal effects of CO_2, temperature, brain region and interaction between CO2 and brain region				
Reference level (Brain region): FB	Estimate	Std. Error	Estimate/ Std. Error	p
(Intercept)	2.93	0.40	7.38	**1.60E-13**
High CO2	-0.02	0.43	-0.05	0.96
Warming	-0.61	0.41	-1.51	0.13
Brain region (FBvsHB)	2.66	0.58	4.60	**4.30E-06**
Brain region (FBvsMB)	4.44	0.75	5.92	**3.20E-09**
High CO2 x Brain region (FBvsHB)	2.31	0.98	2.36	**0.02**
High CO2 x Brain region (FBvsMB)	-0.18	1.04	-0.17	0.86
Reference level (Brain region): MB	Estimate	Std. Error	Estimate/ Std. Error	p

(Intercept)	7.37	0.76	9.67	**<2E-16**
High CO2	-0.20	1.02	-0.20	0.84
Warming	-0.61	0.41	-1.51	0.13
Brain region (MBvsFB)	-4.44	0.75	-5.92	**3.20E-09**
Brain region (MBvsHB)	-1.78	0.88	-2.03	**0.04**
High CO2 x Brain region (MBvsFB)	0.18	1.04	0.17	0.86
High CO2 x Brain region (MBvsHB)	2.49	1.35	1.85	0.06

Numbers in bold p<0.05

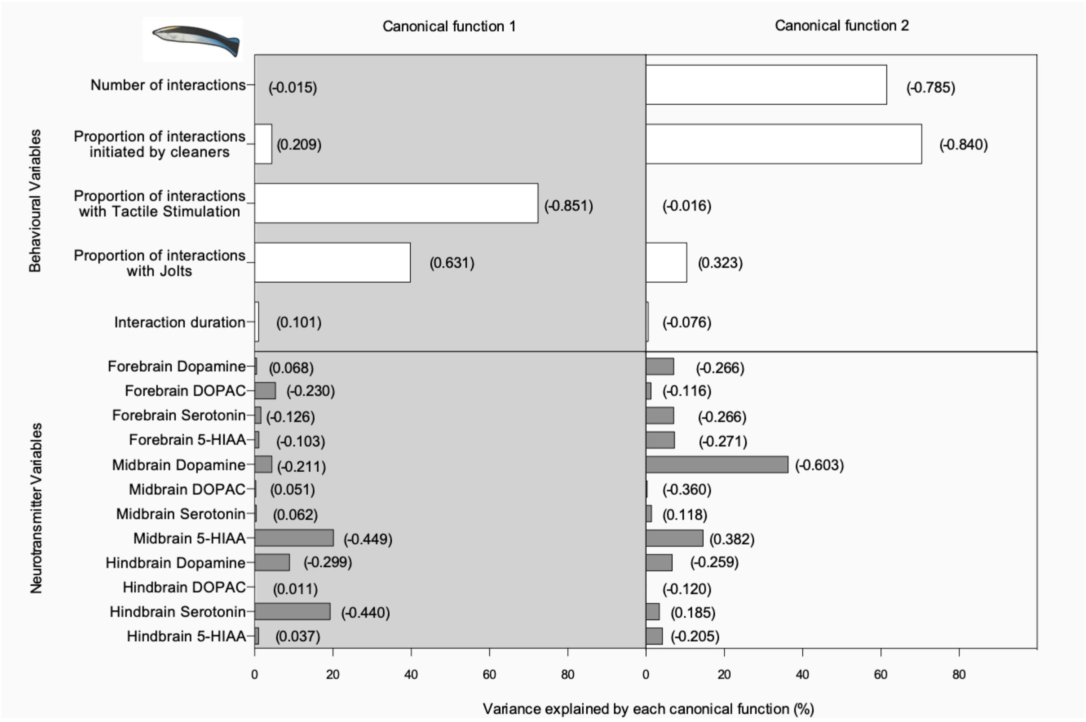

Figure S1. Canonical correlation analysis between behavioural and neurotransmitter variables related to cleaner fish *Labroides dimidiatus*. Percentage of variance explained each canonical function for analysed variables is shown in bars. Numbers in parenthesis indicate respective canonical cross-loading.

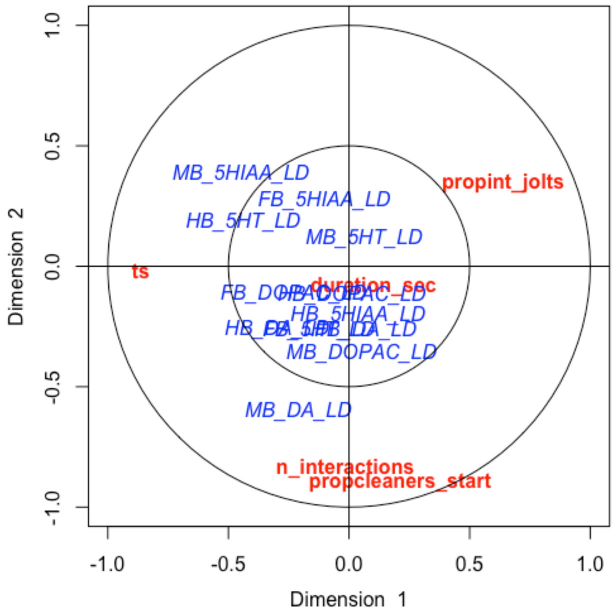

Figure S2. Biplot of the canonical correlation analysis between behavioural and neurotransmitter variables related to cleaner fish *Labroides dimidiatus*.

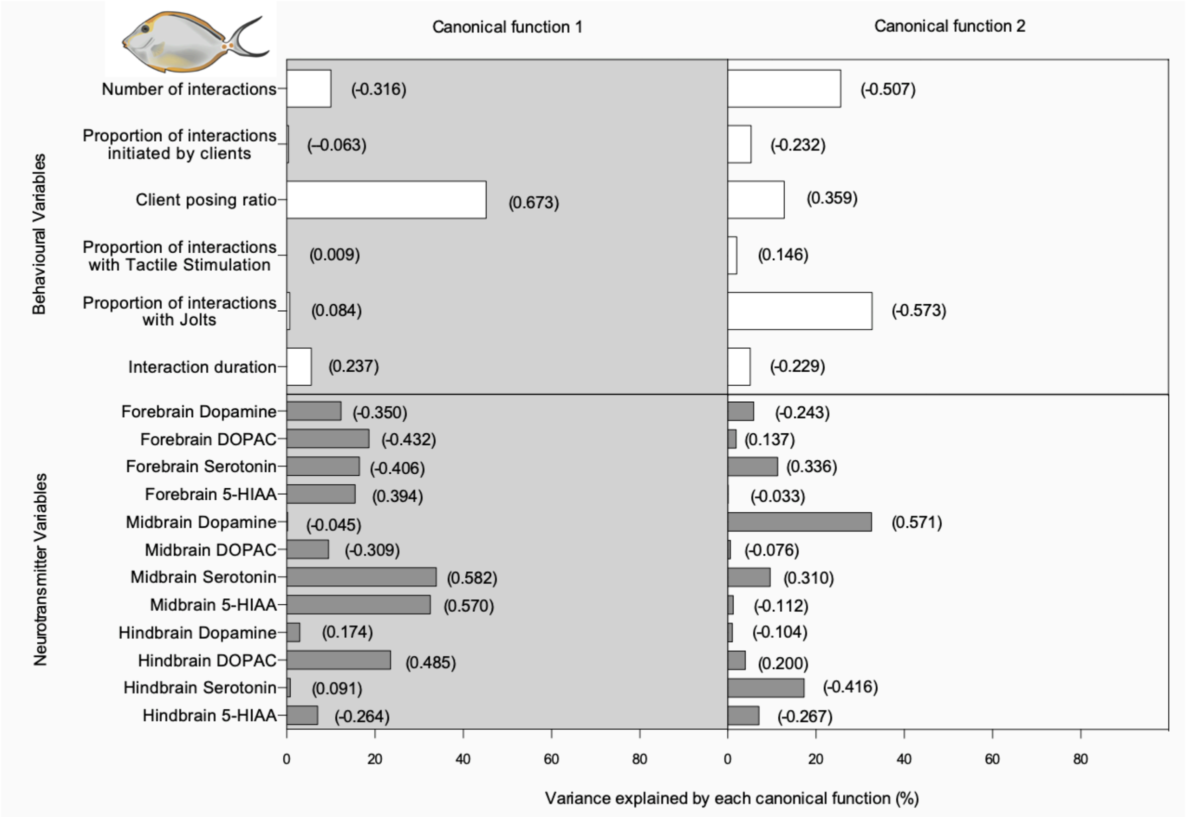

Figure S3. Canonical correlation analysis between behavioural and neurotransmitter variables related to client fish *Naso elegans*. Percentage of variance explained by each canonical function for analysed variables is shown in bars. Numbers in parenthesis indicate respective canonical cross-loading.

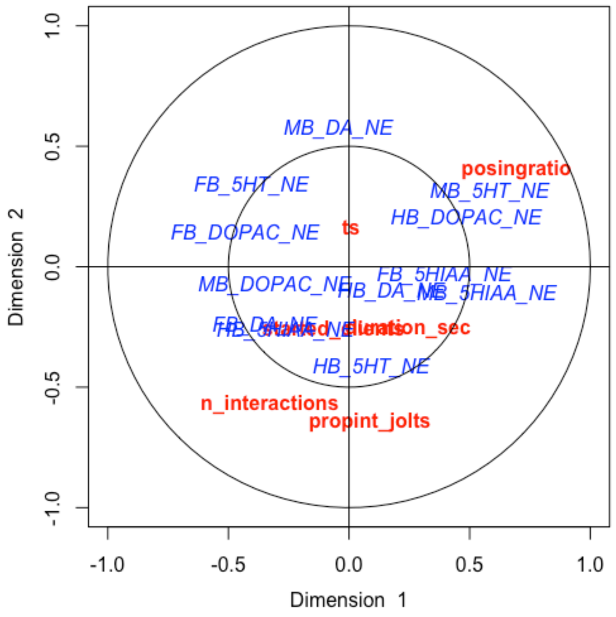

Figure S4. Biplot of the canonical correlation analysis between behavioural and neurotransmitter variables related to client fish *Naso elegans*.
